# Special Issue: Evaluation of the Antitumor Mechanism of Armed Antibodies

**DOI:** 10.3390/ph16121690

**Published:** 2023-12-05

**Authors:** Yoshikatsu Koga, Hiroki Takashima, Shigehiro Koganemaru

**Affiliations:** 1 Department of Strategic Programs, Exploratory Oncology Research & Clinical Trial Center (EPOC), National Cancer Center, Kashiwa 277-8577, Japan; 2 Division of Developmental Therapeutics, Exploratory Oncology Research & Clinical Trial Center (EPOC), National Cancer Center, Kashiwa 277-8577, Japan; hitakash@east.ncc.go.jp; 3 Department of Experimental Therapeutics, National Cancer Center Hospital East, Kashiwa 277-8577, Japan; skoganem@east.ncc.go.jp

This Special Issue focuses on the use of therapeutic antibodies in vitro, in vivo, and in clinical studies. More than 120 years back, Paul Ehrlich dreamt of a “magic bullet” that could specifically attack pathogens [1]. This concept has also been applied to cancer treatment targeting cancer cells without harming normal cells, and a hybridoma technology to produce monoclonal antibodies (mAbs) was developed [2]. Subsequently, “missile therapy” and “immunoconjugates” were easily developed to obtain target-specific mAbs for clinical use in the late 1980s. In the 1990s, antibody engineering technologies that allowed for the genetic modification of murine antibodies to produce chimeric mouse-human antibodies or humanized antibodies, which are less likely to be recognized as foreign antigens by the host immune system and have similar half-lives to those of natural human IgG, were developed. Toward the late 2000s, these mAbs were applied to immunoconjugates again, and were referred to as antibody–drug conjugates (ADCs). ADCs are categorized as armed antibodies and are used to deliver anticancer drugs and radioisotopes. As of August 2020, nine ADCs and one radioimmunotherapy drug have been approved by FDA [3]: gemtuzumab ozogamicin (target: CD33, payload: calicheamicin, specific name: Mylotarg), brentuximab vedotin (CD30, MMAE, Adcetris), trastuzumab emtansine (HER2, DM1, Kadcyla), inotuzumab ozogamicin (CD22, calicheamicin, Besponsa), polatuzumab vedotin (CD79b, MMAE, Polivy), enfortumab vedotin (nectin-4, MMAE, Padcev), trastuzumab deruxtecan (HER2, exatecan derivative, Enhertu), sacituzumab govitecan (TROP-2, SN38, Trodelvy), belantamab mafodotin (CD269, MMAF, Blenrep), and Ibritumomab tiuxetan (CD20, Yttrium-90, Zevalin) [4]. Photoimmunotherapy (PIT) uses armed antibodies that can deliver photoactivating anticancer agents to cancer cells [5]. Bispecific antibodies (BsAbs), especially T cell-engaging bispecific antibodies (T-BsAbs), are also categorized as armed antibodies that can help T cells infiltrate cancer tissues by binding T cells to cancer cells [6]. Chimeric antigen receptor (CAR)-T cells, which genetically express cancer-specific antibodies on T cells, have also been broadly classified as armed antibodies [7]. Thus, armed antibodies are one of the most promising areas of study in cancer therapeutics.

This Special Issue consists of four original and three review articles to provide a comprehensive report on armed antibodies, including ADCs, PIT, BsAbs, and CAR-T cells.

The first article (contribution 1) reported a novel anti-CD73-ADC antibody targeting glioblastomas and focused on the production of the antibody using a unique method. The development of antibodies within the native structure of membrane proteins with multiple transmembrane domains is challenging because it is difficult to prepare antigen with native structures. However, this study produced rat antibody clones using an exosome immunization approach and performed immunoprecipitation using magnetic beads to identify the antigen as CD73. The anti-CD73-ADC exerts an antitumor effect in glioblastoma cell lines based on the expression of CD73. Therefore, these ADCs have the potential to treat cancers with high CD73 expression levels. In addition, this strategy can be used to determine the antigen of any antibody produced by exosome immunization, which may allow this antibody to be developed for other novel antibody therapies.

The second article (contribution 2) was reported to be a novel anti-somatostatin receptor 2 (SSTR2)-ADC for meningiomas. SSTR2s are highly expressed in most meningiomas, but no effective therapy targeting their use to control meningiomas has been approved yet. Therefore, this study developed and evaluated an anti-SSTR2-ADC to target and treat meningiomas. The anti-SSTR2 mAb had a high binding rate of >98% to meningioma cells but a low binding rate of <5% to the normal arachnoidal cells, according to flow cytometric analysis. This fluorescent-labeled ADC targeted and accumulated in meningioma xenografts but not normal organs. The in vitro anticancer cytotoxicity indicated the high potency of these ADCs, with an IC_50_ value of <10 nM. In vivo, the anti-SSTR2 ADC exhibited antitumor efficacy by effectively inhibiting tumor growth at doses of 8 and 16 mg/kg bodyweight. This study demonstrated that the anti-SSTR2 ADC could effectively target meningiomas and reduce tumor growth.

The third article (contribution 3) reported the use of site-specific antibody conjugation for ADCs. Site-specific antibody conjugation generates homogeneous ADCs with high therapeutic indices. However, there are limited examples of site-specific conjugates with a drug-to-antibody ratio (DAR) greater than two, especially those using engineered cysteines. This study designed and introduced free cysteine residues into various CH_2_ and CH_3_ regions in antibodies, based on available Fc structures, to explore and expand site-specific antibody conjugation for ADCs. Among the 38 double-cysteine mutants, 36 displayed comparable expression, with low aggregation similar to the wild-type antibody. PEGylation screening identified 17 double-cysteine mutants with a good conjugation ability and high selectivity. PEGylation was demonstrated to be a valuable and efficient approach to quickly screen mutants with high selectivity and conjugation efficiency. This study demonstrated the feasibility of generating antibody conjugates with a DAR greater than 3.4 and high site-selectivity. The top single- or double-cysteine mutants identified can potentially be applied to site-specific antibody conjugation with cytotoxins or other therapeutic agents as a next-generation conjugation strategy.

The fourth article (contribution 4) reported an evaluation method for PIT. This study aimed to investigate the fluorescence intensity and antitumor effect of PIT using real-time fluorescence observations of tumors, and to predict the required irradiation dose. IR700 showed a sharp decrease in fluorescence intensity in the early stages of treatment and almost reached a plateau at an irradiation dose of 40 J/cm. A significant antitumor effect was observed for irradiation at 40 J/cm, compared with no irradiation; there was no significant difference in the antitumor effect induced by irradiation at 40 and 100 J/cm. These results suggested that the rate of decay of the tumor fluorescence intensity correlated with the antitumor effect of real-time fluorescence imaging during PIT. In addition, when the fluorescence intensity of the tumor plateaued during real-time fluorescence imaging, it was assumed that the laser dose was necessary for treatment.

The first review (contribution 5) assessed the past, present, and future of clinically applied CAR-T cell therapies. First, various clinical trials conducted before the clinical application of CD19-targeted CAR-T cell therapies were summarized. Second, the accumulated real-world evidence and barriers associated with applying the findings of clinical trials to clinical practice from the perspective of quality and the technical aspects were discussed. After providing an overview of all the moving parts involved in the production of CAR-T cell therapies, the characteristics of immune cells and the relationship between immune cells and patients’ lifestyles, including diet and exercise, were discussed. Lastly, future trends in the development of immune cell therapies were highlighted.

The second review (contribution 6) assessed T-BsAbs. As a breakthrough in immunotherapy, T-BsAbs are promising antibody therapies for various types of cancers. In general, T-BsAbs have dual-binding specificity to a tumor-associated antigen and the CD3 subunit, forming a complex with the T cell receptor. This enables T-BsAbs to cross-link tumor cells and T cells, inducing T cell activation and subsequent tumor cell death. Unlike immune checkpoint inhibitors, T-BsAbs serve as T cell activators by stimulating their immune responses via CD3 engagement. Therefore, they can actively redirect host immunity toward tumors, including T cell recruitment from the periphery to the tumor site and immunological synapse formation between tumor cells and T cells. Although the low immunogenicity of solid tumors has increased the challenges in cancer immunotherapy, T-BsAbs capable of immune redirection can greatly benefit patients with such tumors. To investigate the detailed relationship between T-BsAb delivery and T cell redirection, it is necessary to determine how T-BsAbs promote antitumor immunity at the tumor site and stimulate tumor cell death. This review discussed the properties of T-BsAbs (pharmacokinetics, the redirection of anticancer immunity, the local mechanisms of action within tumors), and the challenges in expediting T-BsAb development.

The third review (contribution 7) focused on BsAbs. A breakthrough in the armamentarium of anticancer agents was the introduction of mAbs, which can inhibit aberrantly activated pathways or trigger antigen-specific immune responses. Nonetheless, mAb-mediated targeting often fails due to escape mechanisms, especially antigen loss/downregulation, ultimately resulting in resistance to the therapy. Hence, BsAbs have been developed to target multiple antigens at the same time and facilitate cancer-immune cell interactions, and are being tested in clinical trials, yielding variable safety and efficacy results based on target selection and structure. For hematologic cancers, the BsAb blinatumomab recently acquired FDA approval for B cell acute lymphoblastic leukemia. However, BsAbs used in solid tumors face considerable challenges, such as target antigen selection, biodistribution, and the presence of an immunosuppressive tumor microenvironment. This review focused on the state-of-the-art technology, design, and exploitation of BsAbs against tumors, delineating their mechanisms of action, major pitfalls, and future directions.

This Special Issue highlights the recent findings on armed antibodies and new cancer treatment strategies that incorporate a variety of technologies, including antibodies, linkers, payloads, photosensitizers, and genetically modified T cells. Considering the diverse areas of research, the importance of multidisciplinary teams that use the expertise of basic medical, pharmaceutical, and engineering researchers, as well as clinicians, is indisputable. As new ADCs and PITs continue to be approved, the field of armed antibodies continues to evolve. This Special Issue seeks to encourage the development of new armed antibody technologies for cancer therapy. As the Special Issue “Evaluation of the Antitumor Mechanism of Armed Antibodies 2023” continues, we hope that more novel findings will be submitted.
